# Impact of renal sinus protrusions on achieving trifecta in robot‐assisted partial nephrectomy

**DOI:** 10.1002/bco2.244

**Published:** 2023-04-26

**Authors:** Yuto Hattori, Takanari Kambe, Yuta Mine, Hiroki Hagimoto, Hidetoshi Kokubun, Yohei Abe, Daisuke Yamashita, Naofumi Tsutsumi, Shigeki Arizono, Toshinari Yamasaki, Mutsushi Kawakita

**Affiliations:** ^1^ Department of Urology Kobe City Medical Centre General Hospital Kobe Japan; ^2^ Department of Pathology Kobe City Medical Centre General Hospital Kobe Japan; ^3^ Department of Diagnostic Radiology Kobe City Medical Centre General Hospital Kobe Japan

**Keywords:** anatomy, carcinoma, cross‐sectional, multidetector computed tomography, nephrectomy, renal cell, robotic surgical procedure

## Abstract

**Objective:**

The objective of this work is to assess the relationship between the morphological characteristics of a central tumour and the perioperative outcomes of robot‐assisted partial nephrectomy (RAPN).

**Subjects and Methods:**

We retrospectively analysed the data from 186 patients with central tumours involving the renal sinus, who underwent RAPN in a single‐centre study between February 2015 and June 2022. All cases were assigned a RENAL nephrometry score based on preoperative images. The shape of the protruding portion of the tumour was classified into four types: ‘flat’, ‘spherical’, ‘single‐hump’, and ‘complex‐hump’, and was independently assessed by two readers. The trifecta is defined as the warm ischemia time within 25 min, negative surgical margins, and no major postoperative complications. Univariate and multivariate analyses were performed to identify the factors associated with the failing trifecta.

**Results:**

Trifecta was achieved in 113 cases (60.8%), and the achievement rate in flat, spherical, single‐hump, and complex‐hump types was 83.3%, 74.5%, 64.3%, and 21.3%, respectively. Prolonged warm ischemia time was the primary cause of the failure to achieve the trifecta. The rate of positive surgical margins and upstage to pathological T3a was greater for complex humps while the rate of major complications and postoperative GFR preservation did not differ between shapes. On multivariate analysis for failing trifecta achievement, the complex‐hump protrusion was found to be an independent positive predictor (odds ratio: 15.8; *p* < 0.001), whereas the height and width of protrusion were not significantly related.

**Conclusions:**

The degree of difficulty varied among central tumours, and it was not possible to precisely measure it with existing scoring systems. Complex‐hump protrusions strongly correlate with failure to achieve the trifecta. Preoperative assessment of the morphology of protrusion is useful for predicting outcomes.

## 1 INTRODUCTION

Partial nephrectomy (PN) is the gold standard of treatment for small renal masses as it not only results in better functional outcomes but also has comparable oncological outcomes with those of radical nephrectomy (RN).^1^ For resection and reconstruction, the advantages of robotic surgery include a high‐resolution 3D display, small wrist instruments, and accurate movement through tremor filtration; hence, robot‐assisted partial nephrectomy (RAPN) has gained widespread use. A systematic review similar to our previous study has shown that RAPN results in better perioperative outcomes than laparoscopic PN.^2^ It has also been reported that RAPN can be safely performed on large or highly complex tumours.^3^, ^4^, ^5^

The RENAL nephrometry score and PADUA classification,^6^, ^7^ which are scoring systems based on anatomical findings, are frequently used to assess tumour complexity. The scoring systems have been shown to be associated with the perioperative outcomes following PN.^8^ In both models, the proximity of the tumour to the renal sinus is a common component of each. Some studies regarding the individual components of the RENAL nephrometry score reported that the proximity of the tumour to the renal sinus is crucial.^9^, ^10^ Similarly, in the SPARE score, which simplifies the PADUA classification, renal sinus involvement is scored higher than other factors, suggesting that they have a significant effect on surgical difficulty.^11^, ^12^

Central tumours are considered those that involve the renal sinus. The extent of renal sinus involvement varies from only slightly in contact with the renal sinus to markedly protruding into the renal sinus. Although the degree of protrusion into the renal sinus affects the difficulty of PN, none of the scoring systems take this point into account. Furthermore, there is no consensus on how to adequately evaluate renal sinus protrusion. In this cohort, we assessed the influence of renal sinus protrusion on the perioperative outcomes of RAPN for central tumours.

## 2 SUBJECTS AND METHODS

### 2.1 Study design

Between February 2015 and June 2022, 326 patients underwent RAPN for renal cell carcinoma (RCC) (cT1‐2N0M0) in the single tertiary referral centre. Central tumours, defined as tumours involving the renal sinus,^13^, ^14^ were included in this study. Of 195 central tumours, computed tomography (CT) without contrast medium (*n* = 8) and multiple tumour resections with a single artery clump (*n* = 1) were excluded. Finally, 186 patients with central tumours were included in this study. This study was approved by the Institutional Review Board of Kobe City Medical Centre General Hospital (approval number: zn221019).

**FIGURE 1:**
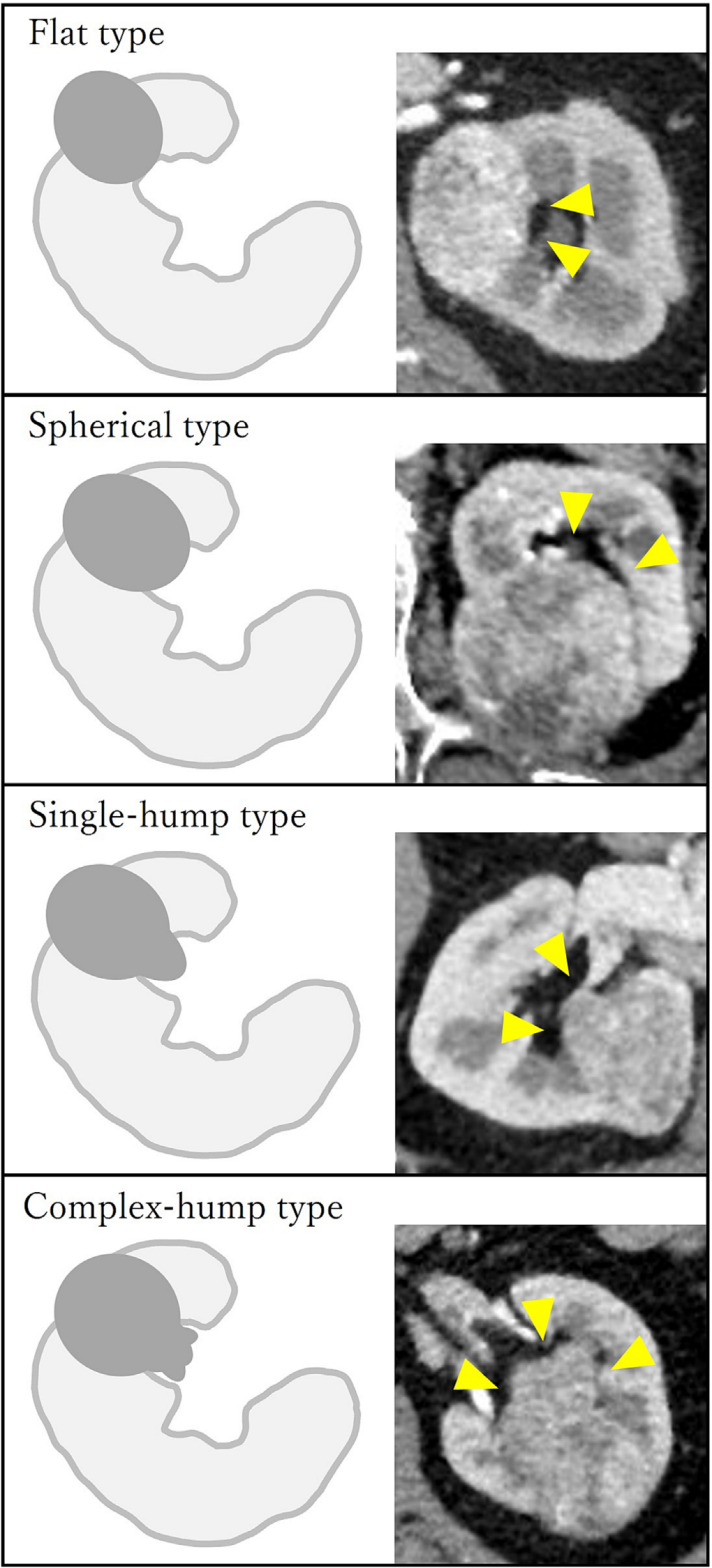
Classification of the shape of tumour protrusions using schemas and CT images (arrowhead shows the protruded part of the tumour). (A) Flat type. (B) Spherical type. (C) Single‐hump type. (D) Complex‐hump type.

All patients underwent contrast‐enhanced CT preoperatively; a total of 182 cases (97.8%) and 171 cases (91.9%) were performed with dynamic contrast and <2 mm thin slices, respectively. As the definition of central tumour, ‘N’ in the RENAL nephrometry score was assigned 3 points in all cases. The protruding shapes were classified into four types: (1) No protrusion (‘flat type’), (2) raised with a spherical shape (‘spherical type’), (3) single protrusion (‘single‐hump type’), and (4) other shapes (e.g., multiple/branched/stemmed/tumour mostly in renal sinus) (‘complex‐hump type’) (Figure 1). All cases of protruding shapes were independently reviewed and classified by a urologist and a radiologist. If the classification differed, re‐evaluation by multiplaner reconstruction and discussion were made before the final classification.

The extracted preoperative characteristics include demographic data (age, gender, body mass index [BMI], Charlson comorbidity index) clinical characteristics of patients (tumour size, glomerular filtration rate [GFR], chronic kidney disease stage according to the kidney disease: Improving Global Outcomes criteria, RENAL nephrometry score), and radiological features of the protruding portion of the tumour (height, width, and shape) (Figure S1). All patients were staged according to the TNM system of the 8th edition of the American Joint Committee on Cancer staging manual.^15^

The perioperative characteristics (operating time, estimated blood loss, type of ischemia, warm ischemia time [WIT], transfusion, major complications, GFR preservation rate), oncological outcomes, and pathological findings were summarized. Oncological outcomes were evaluated in only patients without previous history of RCC.

### 2.2 Surgical technique

A single expert surgeon who had performed over 300 pure laparoscopic partial nephrectomies operated on or supervised in all cases of this study. The approach was determined based on the location of the tumour. Ureteral catheters were not inserted in all cases. After clamping the artery at the hilum and confirming by Doppler ultrasound that blood flow to the kidney ceased, tumour resection commenced. Our procedure is defined as hybrid enucleation according to the Surface–Intermediate–Base margin score (1–1–0).^16^ When the sinus was opened, inner running suturing was performed using a 3‐0 barbed suture (V‐Loc™; Covidien, Ireland). Haemostasis was achieved by monopolar soft coagulation (VIO300D or VIO3, ERBE, Germany) and fibrin‐based haemostat (TachoSil®; CSL Behring, Japan). Parenchymal renorrhaphy was not performed.^17^ All cases underwent CT within 7 days after surgery to monitor for postoperative complications.

### 2.3 Study objectives

The perioperative outcomes were evaluated based on the achievement of the trifecta, which consisted of WIT within 25 min, negative surgical margins, and no major (Clavien–Dindo classification ≥3) postoperative complications within 30 days after surgery.

### 2.4 Statistical analysis

Descriptive statistics are reported as medians and interquartile ranges (IQR) for continuous variables and frequency and proportion for categorical variables. Univariate and multivariate analyses were used to assess the impact of tumour morphology of renal sinus protrusions on failing trifecta achievement. Statistical analyses were performed using EZR, version 1.53. All tests were two‐sided, and statistical significance was considered at *p* < 0.05.

## 3 RESULTS

The demographic and clinical characteristics of all patients are detailed in Table 1. The median tumour size was 3.5 cm (IQR, 2.6–4.5), and the median GFR was 66.0 (IQR, 55.3–77.0). For the shapes of the protrusions, 42 (22.6%), 55 (29.6%), 42 (22.6%), and 47 (25.3%) were of the flat, spherical, single‐hump, and complex‐hump types, respectively.

**TABLE 1:** Demographic and clinical characteristics of all patients.

Number of patients	186	
Median age, years (IQR)	67	(58–76)
Males (%)	127	(68.3)
Median BMI, kg/m^2^ (IQR)	24.6	(21.9–26.5)
Median Charlson comorbidity index (IQR)	2	(2–3)
Number of right kidneys (%)	68	(36.6)
Median tumour size, cm (IQR)	3.5	(2.6–4.5)
Median height of sinus protrusion, cm (IQR)	0.3	(0.1–0.8)
Median width of sinus protrusion, cm (IQR)	1.4	(0.8–2.1)
Shape of sinus protrusion, number (%)		
Flat	42	(22.6)
Spherical	55	(29.6)
Single hump	42	(22.6)
Complex hump	47	(25.3)
Median preoperative GFR, mL/min/1.73 m^2^ (IQR)	66.0	(55.3–77.0)
Number of solitary kidney and/or CKD stage ≥3 (%)	63	(33.9)
RENAL nephrometry score, number (%)		
6	9	(4.8)
7–9	145	(78.0)
10–12	32	(17.2)

Abbreviations: BMI, body mass index; CKD, chronic kidney disease; GFR, glomerular filtration rate; IQR, interquartile range.

The trifecta was achieved in 113 cases (60.8%) and the median WIT was 22 min (IQR, 15–29). Eight cases developed major complications, including three cases of urinary leakage, two cases of pseudoaneurysm, two cases of cholecystitis, and one case of inferior mesenteric artery (IMA) bleeding. IMA bleeding was discovered 3 days after RAPN and required interventional radiology. No case was converted to RN or open surgery. Histologically, 181 cases were diagnosed with RCC, and 157 were of the clear‐cell subtype. Upstaging to pathological stage T3a was observed in eight cases; perirenal fat, venous, and renal sinus invasions were detected in two, three, and three cases, respectively. Additionally, 15 cases (8.1%) had positive surgical margins, three of which had tumour exposure in the protruding portion (Table S1). WIT prolonged as the shape of the protrusion became more complex, although the postoperative GFR preservation rate was maintained at nearly 90% regardless of shape. The major complication rate was not associated with the shape of protrusion, while the prevalence of positive surgical margins and pathological upstaging to T3a were greater in tumours with complex humps (Table 2).

**TABLE 2:** Relationship between the shape of protrusion and RENAL nephrometry score, the trifecta achievement rate, the GFR preservation rate, and the upstaging to pathological T3.

	Total (*n* = 186)		Flat (*n* = 42)		Spherical (*n* = 55)		Single‐hump (*n* = 42)		Complex‐hump (*n* = 47)	
**Number of RENAL nephrometry score (%)**										
6	9	(4.8)	4	(9.5)	3	(5.5)	2	(4.8)	0	(0)
7–9	145	(78.0)	33	(78.6)	45	(81.8)	36	(85.7)	31	(66.0)
10–12	32	(17.2)	5	(11.9)	7	(12.7)	4	(9.5)	16	(34.0)
Number of trifecta achievement(%)	113	(60.8)	35	(83.3)	41	(74.5)	27	(64.3)	10	(21.3)
Positive surgical margins	15	(8.1)	0	(0)	3	(5.5)	2	(4.8)	10	(21.3)
Major complication (Clavien–Dindo ≥3)	8	(4.3)	1	(2.4)	2	(3.6)	2	(4.8)	3	(6.4)
WIT >25 min	66	(35.5)	6	(14.3)	11	(20.0)	13	(31.0)	36	(76.6)
**Median preservation rates of GFR (IQR)**										
1 month after surgery	93	(86–100)	93	(86–99)	93	(84–100)	94	(89–102)	91	(85–98)
3 months after surgery	92	(84–100)	92	(81–98)	95	(86–100)	94	(85–99)	88	(81–97)
6 months after surgery	91	(85–100)	92	(85–100)	92	(85–98)	90	(84–100)	90	(84–96)
12 months after surgery	89	(82–97)	89	(82–98)	88	(82–96)	91	(79–97)	90	(84–96)
Number of upstage to pathological T3a (%)	8	(4.3)	0	(0)	1	(0)	0	(0)	7	(14.9)

Univariate analysis demonstrated that achieving trifecta was significantly related to the complex‐hump shape. On multivariate analysis, which was adjusted with each factor of the RENAL nephrometry score, the complex‐hump shape (OR: 15.8, *p* < 0.001) was an independent positive predictor for the failure to achieve the trifecta (Table 3).

**TABLE 3:** Univariable and multivariable logistic regression analyses of factors predicting failure to achieve the trifecta.

Variable		Univariate analysis			Multivariate analysis		
		OR	(95% CI)	*p* value	OR	(95% CI)	*p* value
R of nephrometry score	1	Reference			Reference		
	2	3.36	(1.75–6.45)	<0.001	3.39	(1.46–7.85)	0.004
	3	3.09	(0.78–12.2)	0.108	4.43	(0.81–24.2)	0.086
E of nephrometry score	1	Reference			Reference		
	2	1.06	(0.56–2.03)	0.858	1.48	(0.63–3.47)	0.372
	3	1.44	(0.58–3.60)	0.435	2.31	(0.66–8.03)	0.188
L of nephrometry score	1	Reference			Reference		
	2	0.84	(0.36–1.96)	0.690	0.61	(0.23–1.65)	0.334
	3	1.06	(0.48–2.36)	0.889	0.65	(0.25–1.71)	0.383
Morphology of renal sinus protrusion	Flat	Reference			Reference		
	Spherical	1.71	(0.62–4.70)	0.301	1.62	(0.54–4.83)	0.388
	Single hump	2.78	(0.99–7.77)	0.051	2.58	(0.87–7.7)	0.087
	Complex hump	18.5	(6.34–54.0)	<0.001	15.8	(5.03–49.5)	<0.001

Excluding five patients who were not histologically diagnosed with RCC and three patients with a history of contralateral RCC treated by resection, the median follow‐up period in 178 cases was 35 months (IQR, 20–54). One case had local recurrence, whereas eight developed distant metastases. Six of the eight patients with distant metastases received systemic therapy. There were eight deaths, including one cancer‐specific death. Of the 15 cases with positive surgical margins, there was no case of local recurrence and two cases of distant metastasis.

## 4 DISCUSSION

Central tumours are known to be one of the high‐complexity tumours. Indeed, the trifecta achievement rate and margin negative rate in central tumours were lower than those in noncentral tumours in our institute (60.8% vs. 94.9% and 91.9% vs. 97.4%, respectively). Although many studies have reported the usefulness of the RENAL nephrometry score and the PADUA classification,^8^ these scoring systems cannot distinguish the complexity of central tumours and accurately evaluate the degree of protrusion into the renal sinus. Our study focused on the influence of the degree of tumour protrusion into the renal sinus on the achievement of trifecta during RAPN for central tumours. Among the central tumours, a remarkable difference was detected in the rate of achieving trifecta between the flat (83.3%) and complex‐hump (21.3%) types. Furthermore, multivariate analysis of the preoperative image features showed that other than R score the complex‐hump type was an independent predictor significantly associated with the failure to achieve trifecta (*p* < 0.001).

As variables for evaluating the renal sinus protrusion, we investigated the height, width, and shape of the protrusion part. The height and width not only showed no significant difference in the multivariate analysis but also varied among the readers; hence, they were excluded as inappropriate variables for evaluation (Table S2). On the other hand, the protruding shape can be easily evaluated. Furthermore, it is possible to understand why the protruding shape has a stronger correlation with the difficulty in considering the growth process of the tumour.

Most renal tumours grow in a spherical and expansive pattern within the renal parenchyma. When tumours reach the renal sinus, protrusion into the renal sinus occurs as a single hump, which is presumed to be a result of the difference in the tumour growth rate between the parenchyma and low‐resistance renal sinus fat. As tumours further expand into the gaps of the existing structures, such as blood vessels and the urinary collecting system, the protrusion seems to be complex. Indeed, in complex‐hump types, we sometimes recognize the vessels and the urinary collecting system were embedded in the tumour notch intraoperatively. This may explain why the shape of the protrusion influences the degree of RAPN difficulty.

At our institution, when performing RAPN for a central tumour, an enucleoresection with blunt dissection is performed from the upper to the middle part of the tumour, following which enucleation is performed in the renal sinus. Along with tumour contour, blood vessels and urinary collecting systems are carefully dissected from the tumour. The veins and urinary collecting systems may sometimes stretch and adhere to the tumour, making it difficult to distinguish them from the tumour pseudo‐capsule. In these cases, these structures are reconstructed following sharp dissection with the margins of the pseudo‐capsule. Similarly, in the case of sinus‐fat adhesions, the fat is excised with a tumour. Due to these careful procedures, parenchymal renorrhaphy can be omitted.

As the approach to the renal sinuses is often narrow and deep, an optimal working angle as well as gentle tumour manipulation, careful dissection, and haemostasis are crucial. Although the procedure is time consuming, the preservation of the vessels and urinary collection systems reduces the incidence of complications. Following the contour of the tumour during enucleation prevents incision of the tumour and can be performed in hump‐type protruding tumours. Tumour enucleation is known to be a safe procedure preserving more of the parenchymal vasculature,^18^ and its impact seems to be more pronounced for central tumours. We opine that tumour enucleation is feasible even for central tumours.

WIT was prolonged in 66 of the 73 cases that failed to achieve trifecta. In 52 cases, prolonged WIT was the only cause of failure to achieve the trifecta. Arora et al. reported that wide resection of superficial portions is a useful method to resect buried tumours.^19^ Although widening the resection margins may effectively shorten and simplify the procedure, it may negatively affect renal function. In this study, despite obvious WIT prolongation, renal function was sufficiently preserved. Although the WIT is an important index directly related to the surgical technique for preserving renal function,^20^ the effects of WIT on renal function remain controversial. Anceschi et al. reported that instead of WIT, the preservation rate of the GFR was more relevant to predict renal functional prognosis and overall survival.^21^

Regarding the oncological outcomes at the 35‐month follow‐up, distant metastasis developed in eight cases, and cancer‐related death occurred in one case. Considering that recurrence after RAPN often occurs within 3 years,^22^ these results may seem acceptable; however, it should be noted that complex‐hump types tend to have a high rate of pathological upstaging to T3 and positive surgical margins.

In general, RAPN is not indicated for clinical T3 stage RCC; however, obtaining an accurate preoperative T staging is difficult. Teishima et al. reported that an irregular tumour morphology was associated with an upstaging to T3.^23^ Similarly, in our cohort, the complex‐hump type tended to have more frequent upstaging to T3. Recently, Yim et al. reported that RAPN for clinical T3 tumours resulted in favourable outcomes.^24^ Additionally, Koo reported that for clinical T3 tumours, the oncological outcomes of RAPN were not different from those of RN.^25^ Bertolo et al. reported that in 512 cases that scored 3 points on the ‘N’ score of the RENAL nephrometry score, invasion of renal sinus fat was detected in 24 cases and that these cases had favourable outcomes with negative surgical margins after resection for pathological stage T3 tumours.^26^ The effects of positive surgical margins on oncological outcomes remain equivocal^27^; however, RAPN is technically possible even for select cases with stage pT3 tumours. Further long‐term observation is needed to explore the advantages and disadvantages of RAPN for complex‐hump‐type tumours.

Our study has several limitations. First, it was a single‐institution study with a small sample size that had a retrospective design. Second, the RAPN was performed by experienced surgeons, which may limit the generalizability of our results. Third, it may be difficult to not only distinguish humps from crossing vessels but to also classify the shape of protrusion unless dynamic thin‐slice CT is used.

Despite these limitations, the shape of the protrusion is a novel finding that may be used as an indication for surgical procedures. This study also showed that the difficulty of RAPN for central tumours, which is not stratified by existing scoring systems, varied significantly with the shape of the protrusion. Although RAPN is technically feasible, RAPN for complex‐hump‐type tumours is controversial; hence, clinicians should proceed with caution. Furthermore, considering the increasing use of RAPN for highly complex tumours, a new scoring system may be necessary that accurately assesses renal sinus protrusion.

In conclusion, the degree of difficulty varied among central tumours, and it was not possible to precisely measure it with existing scoring systems. Complex‐hump‐type protrusions strongly correlate with failure to achieve trifecta due to WIT prolongation and surgical margin positivity. Accurate preoperative assessment of the shape of protrusion is necessary for predicting outcomes.

## AUTHOR CONTRIBUTIONS

**Yuto Hattori:** Conceptualization; writing—original draft; preparation; writing—review & editing. **Takanari Kambe:** Data curation; investigation. **Yuta Mine:** Data curation; investigation. **Hiroki Hagimoto:** Data curation; investigation. **Hidetoshi Kokubun:** Data curation; investigation. **Yohei Abe:** Data curation; investigation. **Daisuke Yamashita:** Data curation; investigation; writing—review & editing. **Naofumi Tsutsumi:** Data curation; investigation. **Shigeki Arizono:** Data curation; investigation; writing—review & editing. **Toshinari Yamasaki:** Conceptualization; writing—review & editing. **Mutsushi Kawakita:** Conceptualization; writing—review & editing; supervision.

## CONFLICT OF INTEREST STATEMENT

The authors declare no conflicts of interest.

## Supporting information


**Figure S1.** The base of the renal sinus protrusion was defined as the protrusion width (solid line), and the distance from the greatest extent of protrusion to the base was defined as the protrusion height (dotted line).

**Table S1.** Perioperative and Pathological variables

**Table S2.** Univariate and multivariable logistic regression analyses of the height and width of the renal sinus protrusion for trifecta achievement
