# Supplementary figures and images for: Impact of renal sinus protrusions on achieving trifecta in robot‐assisted partial nephrectomy
Source: BJUI Compass. 2023 Apr 26;4(5):584–90. doi: 10.1002/bco2.244 (PMC10447216; doi:10.1002/bco2.244)

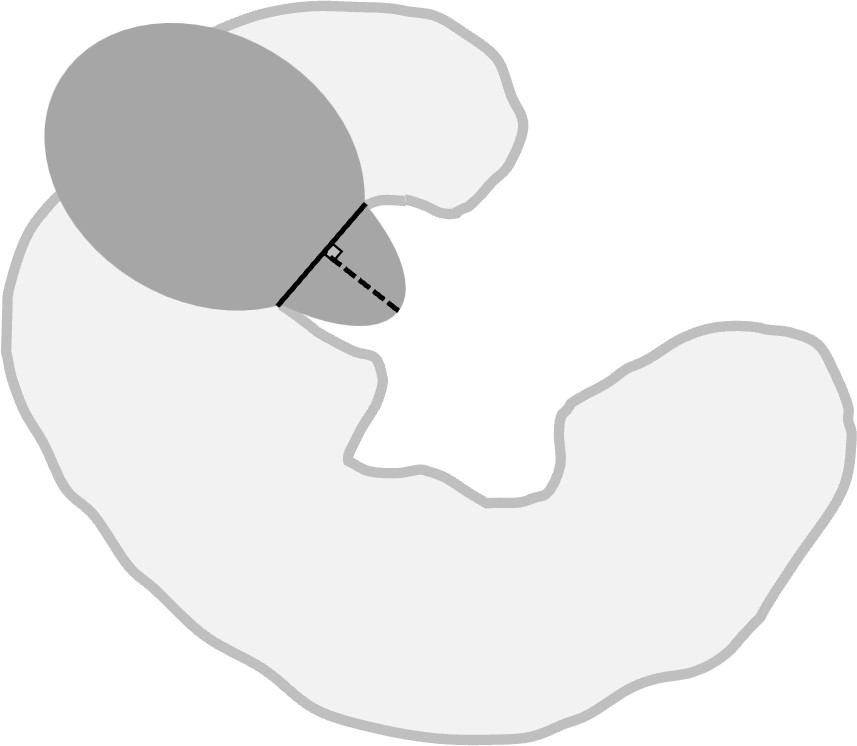

Supplement: Supplementary file 1 — Figure S1. The base of the renal sinus protrusion was defined as the protrusion width (solid line), and the distance from the greatest extent of protrusion to the base was defined as the protrusion height (dotted line). [file BCO2-4-584-s002.jpg]
