# Supplementary material for: Impact of renal sinus protrusions on achieving trifecta in robot‐assisted partial nephrectomy
Source: BJUI Compass. 2023 Apr 26;4(5):584–90. doi: 10.1002/bco2.244 (PMC10447216; doi:10.1002/bco2.244)
Supplement: Supplementary file 2 — Table S1. Perioperative and Pathological variables [file BCO2-4-584-s001.docx]

Supplemental Table 1. Perioperative and Pathological variables

| Median operative time, min (IQR) | | 181 | (159-214) |
| --- | --- | --- | --- |
| Number transperitoneal approach procedures (%) | | 79 | (42.5) |
| Median estimated blood loss, mL (IQR) | | 0 | (0-93) |
| Type of ischemia, number (%) | |  |  |
| Total clamping | | 170 | (91.4) |
| Selective clamping | | 16 | (8.6) |
| Median warm ischemia time, min (IQR) | | 22 | (15-29) |
| Transfusions, number (%) | | 3 | (1.6) |
| Major complications (Clavien–Dindo ≥3), number (%) | | 8 | (4.3) |
| IMA bleeding | → embolization | 1 |  |
| pseudoaneurysm | → embolization | 2 |  |
| urinary leak | → ureteral stenting | 3 |  |
| cholecystitis | → operation | 2 |  |
| Number of patients who achieved trifecta (%) | | 113 | (60.8) |
| Median duration of postoperative stay, days (IQR) | | 6 | (5-7) |
| Histopathology (%) | |  |  |
| Clear cell renal cell carcinoma | | 157 | (84.4) |
| Papillary renal cell carcinoma | | 12 | (6.5) |
| Chromophobe renal cell carcinoma | | 9 | (4.8) |
| Mucinous and spindle cell carcinoma | | 1 | (0.5) |
| Renal cell carcinoma unclassified | | 2 | (1.1) |
| Benign | | 2 | (1.1) |
| Other malignancy | | 3 | (1.6) |
| Pathological T stage*, number (%) | |  |  |
| pT1 | | 167 | (89.8) |
| pT2 | | 6 | (3.2) |
| pT3a | | 8 | (4.3) |
| Grade*, number (%) | |  |  |
| 1–2 | | 138 | (74.2) |
| 3–4 | | 34 | (18.3) |
| Positive surgical margins*, number (%) | | 15 | (8.1) |
| Median follow-up, months (IQR) | | 35 | (20-54) |
| Median preservation rate of GFR (IQR) | |  |  |
| 1 month after surgery | | 93 | (86-100) |
| 3 month after surgery | | 92 | (84-100) |
| 6 month after surgery | | 91 | (85-100) |
| 12 month after surgery | | 89 | (82-97) |
| Number of local recurrence** (%) | | 1 | (0.6) |
| Number of distant metastasis** (%) | | 8 | (4.7) |
| Deaths, number** (%) | |  |  |
| all cause | | 8 | (4.7) |
| due to RCC | | 1 | (0.6) |
| IMA = inferior mesenteric artery; RCC = renal cell carcinoma | | | |

* only for renal cell carcinoma

** only for initially renal cell carcinomas.
