# Supplementary material for: Impact of renal sinus protrusions on achieving trifecta in robot‐assisted partial nephrectomy
Source: BJUI Compass. 2023 Apr 26;4(5):584–90. doi: 10.1002/bco2.244 (PMC10447216; doi:10.1002/bco2.244)
Supplement: Supplementary file 3 — Table S2. Univariate and multivariable logistic regression analyses of the height and width of the renal sinus protrusion for trifecta achievement [file BCO2-4-584-s003.docx]

Supplemental Table 2. Univariate and multivariable logistic regression analyses of the height and width of the renal sinus protrusion for trifecta achievement

|  | | Univariate analysis | | | Multivariate analysis | | |
| --- | --- | --- | --- | --- | --- | --- | --- |
|  |  | OR | (95% CI) | p value | OR | (95% CI) | p value |
| R of nephrometry score | 1 |  | |  | Reference | |  |
|  | 2 |  |  |  | 2.28 | (0.90-5.77) | 0.081 |
|  | 3 |  |  |  | 2.26 | (0.36-14.0) | 0.383 |
| E of nephrometry score | 1 |  | |  | Reference | |  |
|  | 2 |  |  |  | 1.17 | (0.48-2.83) | 0.729 |
|  | 3 |  |  |  | 1.62 | (0.44-5.91) | 0.466 |
| L of nephrometry score | 1 |  | |  | Reference | |  |
|  | 2 |  |  |  | 0.50 | (0.18-1.36) | 0.173 |
|  | 3 |  |  |  | 0.50 | (0.19-1.35) | 0.173 |
| Height of the protrusion to renal sinus (cm) | | 6.95 | (3.38-14.3) | <0.001 | 1.62 | (0.56-4.72) | 0.378 |
| Width of the protrusion to renal sinus (cm) | | 3.01 | (2.01-4.50) | <0.001 | 1.61 | (0.86-3.02) | 0.141 |
| Morphology of  renal sinus protrusion | Flat |  | |  | Reference | |  |
|  | Spherical |  |  |  | 0.98 | (0.30-3.24) | 0.972 |
|  | Single hump |  |  |  | 1.18 | (0.32-4.38) | 0.802 |
|  | Complex hump |  |  |  | 5.06 | (1.07-24.0) | 0.041 |
